# Factors Impacting Treatment Decisions in Caregivers of Autistic Children

**DOI:** 10.3390/bs16081280

**Published:** 2026-07-27

**Authors:** Tiffany G. Harris, Julia Record, Stephanie D. Smith

**Affiliations:** 1 Neurodevelopment and Behavioral Psychology Program, Cincinnati Children’s Hospital Medical Center, Cincinnati, OH 45229, USA; tiffany.harris2@cchmc.org; 2 School of Psychology, The University of Southern Mississippi, Hattiesburg, MS 39406, USA; julia.record@usm.edu

**Keywords:** Common Sense Model, Autism Spectrum Disorder, illness perceptions, coping behaviors, treatment seeking, caregiver decision-making

## Abstract

Leventhal’s Common Sense Model (CSM) is a theoretical framework developed to understand the self-regulatory processes involved in adapting to and managing health threats. This is the first known study to examine all components of the CSM (i.e., illness perceptions and coping behaviors) in its application to treatment seeking behaviors in caregivers of autistic children (*N* = 288). Results revealed a significant indirect pathway from caregivers’ perceptions of the unpredictable nature of ASD symptoms to intentions to seek treatment for their children through problem-focused coping (β = 0.07, *p* = 0.02, 95% CI [0.01, 0.13]). Additionally, caregivers’ perceptions about the controllability of their children’s ASD symptoms were positively related to treatment seeking behaviors (β = 0.19, *p* = 0.03, 95% CI [0.06, 0.34]). These findings suggest that providers assess and then use CBT-based strategies (e.g., cognitive restructuring) to potentially modify aspects of caregivers’ cognitions and coping behaviors during ASD feedback sessions to promote prompt treatment seeking for their autistic children.

## 1. Introduction

Autism Spectrum Disorder (ASD) is a neurodevelopmental disorder characterized by differences in how children socially interact and communicate with others. These social differences are often accompanied by restricted interests and repetitive patterns of behaviors (American Psychiatric Association, 2013). ASD is considered a lifelong neurodevelopmental difference, but early engagement in supportive services has been shown to positively influence prognosis, such as increasing cognitive, language, and adaptive skills (Dawson et al., 2010; Elder et al., 2017; Evangelou et al., 2025; Vivanti et al., 2019). Given the benefits of early intervention services, it is vital to identify factors that influence the decision-making of caregivers concerning their use of supportive services for their autistic children. As such, the purpose of this study was to evaluate and apply a well-established theoretical model from the chronic illness literature (i.e., Leventhal’s Common Sense Model of Self-Regulation) to caregivers of autistic children to better understand how caregivers make sense of an autism diagnosis and how these beliefs shape their own coping behaviors and the treatment decisions they make.

Leventhal’s Common Sense Model of Self-Regulation (the CSM; Leventhal et al., 1980) was developed to understand how lay perceptions of a chronic illness diagnosis influence symptom management (Leventhal et al., 1980). The CSM proposes that coping behaviors mediate the pathway between cognitive and emotional perceptions of a diagnosis and symptom outcomes. According to the CSM, cognitive representations of a chronic illness diagnosis include concepts such as beliefs about, causes of, and personal control over the symptoms of the diagnosis; consequences of the diagnosis; and knowledge about the duration or timeline of the condition. Emotional representations are theorized to encompass negative emotional responses to a diagnosis, such as fear, anger, and worry (Leventhal et al., 2016). Importantly, cognitive perceptions of a chronic illness diagnosis have been shown to be amenable to change, especially with ongoing exposure to and management of the diagnosis (Leventhal et al., 2001). The malleability of these cognitive perceptions makes them an optimal target for intervention, especially if the cognitive perceptions are inaccurate or unhelpful to the person with the chronic illness (Leventhal et al., 2001). Besides the cognitive and emotional perceptions of a diagnosis, the CSM specifies two additional factors that constitute the ongoing self-regulation process of managing a chronic illness diagnosis: coping behaviors and the appraisal of illness outcomes (Leventhal et al., 2001). Coping behaviors include the plans or tactics used to treat or control the health threat, which can be either adaptive (e.g., adhering to a medication regimen, attending doctor’s appointments) or maladaptive (e.g., denial of the diagnosis, avoidance of appointments or initiating treatments; Leventhal et al., 2001). Leventhal et al. (2001) suggest illness outcomes are the consequences of these adaptive or maladaptive coping behaviors (e.g., a reduction in high blood pressure with consistent use of medication). The CSM has been used to understand patient adjustment and self-management for a wide variety of chronic illnesses. Indeed, studies have shown that coping behaviors mediate the association between illness perceptions and illness outcomes (e.g., Hagger et al., 2017). Specifically, certain cognitive illness perceptions (i.e., illness is understandable, controllable, has harmful consequences) are positively linked to adaptive coping strategies (i.e., cognitive appraisal; Richardson et al., 2017), which in turn lead to positive illness outcomes (e.g., mental well-being). Alternatively, certain cognitive illness perceptions (i.e., illness is unpredictable) and emotional illness perceptions (i.e., negative emotions) are positively linked to maladaptive coping strategies (i.e., avoidance, denial; Richardson et al., 2017), which in turn lead to negative illness outcomes (e.g., anxiety, psychological distress; Carver et al., 1989; Chen et al., 2024; Hagger et al., 2017).

The CSM has more recently been applied to understand caregivers’ cognitive and emotional perceptions of their children’s ASD diagnosis. Although not a chronic illness, ASD is conceptualized as a lifelong neurodevelopmental difference, and many autistic children benefit from supportive services throughout the lifespan (Shea & Mesibov, 2009). Thus, the CSM may be helpful in elucidating how caregivers process and respond to their children’s ASD diagnosis. In fact, Mire et al. (2018) sought to determine whether caregivers’ illness perceptions of their children’s ASD comprised similar domains as those proposed by the CSM. Using a revised questionnaire for caregivers with autistic children that was based on a well-established measure of cognitive and emotional illness perceptions among patients with chronic illness (i.e., the Illness Perceptions Questionnaire-ASD), Mire et al. (2018) found that a 5-factor solution best fit their data (rather than the 6-factor solution found in the chronic illness literature) and items loaded as expected onto subscales capturing illness timeline, illness consequences, illness coherence, and emotional representations. However, the items comprising the personal control subscale and the treatment control subscale loaded onto a single factor, suggesting that caregivers may not conceptualize control over their children’s treatment as separate from control over ASD symptoms (Mire et al., 2018). They also found that caregivers tend to have more negative emotions about their children’s diagnosis when they view ASD as chronic, uncontrollable, not easily understood, and resulting in serious consequences (e.g., financial, social, behavioral; Mire et al., 2018). Additionally, caregivers who view ASD as causing serious consequences tend to view ASD as chronic while caregivers who endorse understanding ASD tend to report having greater control over ASD symptoms (Mire et al., 2018). Overall, these findings provide evidence that the cognitive and emotional perceptions of ASD held by caregivers map onto the cognitive and emotional perceptions proposed by the CSM.

To our knowledge, no known studies have examined cognitive and emotional perceptions of ASD and how they relate to coping behaviors in caregivers of autistic children; however, a few studies have examined caregiver cognitive and emotional illness perceptions of ASD in relation to outcomes such as treatment decisions and caregiver stress. Specifically, caregivers who select evidence-based educative treatments (e.g., applied behavioral analysis) tend to report a greater degree of serious consequences associated with ASD (Al Anbar et al., 2010). In contrast, caregivers are less likely to pursue evidence-based educative treatments when they report increased negative emotions about their children’s ASD (Al Anbar et al., 2010) and when they have strong beliefs that their children’s ASD is caused by early traumatic experiences (Dardennes et al., 2011). The use of psychopharmaceutical treatments is more common in caregivers who view ASD as unpredictable in nature (Al Anbar et al., 2010) and when the cause of ASD is thought to be attributed to maternal illness during pregnancy (Dardennes et al., 2011); the use of psychopharmaceuticals is less likely in caregivers who perceive a greater degree of personal control over ASD symptoms (Al Anbar et al., 2010). Alternatively, caregiver beliefs that ASD is caused by a food allergy or a chemical imbalance in the brain is associated with the use of special diets and vitamins (Al Anbar et al., 2010) while caregivers who attribute environmental causes (e.g., toxins in vaccines, environmental pollutants) to ASD are more likely to be vaccine hesitant (Chang & Goin-Kochel, 2020; Goin-Kochel et al., 2020; Mensah-Bonsu et al., 2021; Sahni et al., 2020). Finally, caregivers reporting more negative emotional illness perceptions about ASD report higher levels of stress related to caring for their children (Mire et al., 2022) whereas caregivers reporting higher perceived control over ASD endorse better family quality of life (Papadopoulos et al., 2023). In essence, studies applying the CSM to caregivers of autistic children have found significant links between certain caregiver perceptions of ASD and a limited number of distal outcomes (i.e., type of treatment, caregiver stress).

Although there is a growing body of work looking at associations between illness perceptions and more distal outcomes (e.g., treatment decisions, caregiver stress), the coping behaviors that might help explain these associations have not been evaluated in studies applying the CSM to caregivers of autistic children. Similar to illness perceptions, coping behaviors are also malleable to intervention (Zhao et al., 2022) so understanding coping behaviors that might interfere or promote the adoption of evidence-based treatment is important and will better equip providers to intervene and teach caregivers strategies to increase the likelihood of positive developmental outcomes for their children. As such, the primary aim of this study is to comprehensively evaluate the applicability of the entirety of the CSM (i.e., cognitive and emotional perceptions of ASD, coping behaviors, and treatment outcomes) in caregivers of autistic children. Specifically, it was examined whether cognitive and emotional illness perceptions predicted caregivers’ treatment seeking behaviors through coping behaviors. Given the findings of studies evaluating the CSM in the chronic illness and autism literature, it was hypothesized that certain cognitive illness perceptions (i.e., autism is controllable, understandable, has harmful consequences) of ASD will lead to increased problem-focused coping strategies, which will then lead to increased treatment seeking behaviors. Conversely, it is hypothesized that certain cognitive illness perceptions (e.g., autism is unpredictable) and emotional illness perceptions (i.e., autism elicits negative emotions) of ASD will lead to increased active-avoidance coping strategies, which will then lead to reduced treatment seeking behaviors. Importantly, a better understanding of how caregiver cognitive and emotional perceptions of ASD influence each other and relate to coping behaviors and treatment seeking behaviors may help guide best practices for diagnostic feedback and intervention planning so the impact of receiving an ASD diagnosis is attenuated and treatment seeking is promoted.

## 2. Materials and Methods

### 2.1. Participants

Participants were eligible for this study if they identified as a caregiver of an autistic child who was aged 17 years or younger. Participants were recruited from four private practices or centers specializing in ASD assessment located in the southeastern or southcentral regions of the US or through a crowd-sourcing website (Prolific). Forty-four participants accessed the study survey from in-person recruitment sites and 18 (40%) completed the survey in its entirety. A total of 2000 caregivers completed a study screener through Prolific and 349 of those caregivers were found to be eligible for the study and were invited to participate. The study survey was completed by 279 participants (80%) and 9 participants’ data were not used in study analyses as they reported their children’s age to be 18 years or older or they failed two of three attention checks. This resulted in a final sample size of 288 participants. Caregiver and child demographic characteristics along with child diagnostic and treatment information are presented in Table 1 and Table 2. Caregivers were 41 years old on average, and most were biological parents of the child, identified as White women, were married, college-educated, and employed full-time. Caregivers reported that their children were 9 years old on average at the time of survey completion and 5 years of age on average when they received diagnoses of ASD. Children were mostly White boys diagnosed with ASD by a psychologist or medical professional and were described as having low to moderate support needs.

### 2.2. Measures

The Illness Perception Questionnaire–Revised, Autism Spectrum Disorder (IPQ-R-ASD; Mire et al., 2017) was used to measure caregiver’s perceptions about their children’s ASD diagnosis. The IPQ-R-ASD is a 76-item self-report questionnaire that measures dimensions of illness perceptions according to Leventhal’s CSM (Leventhal et al., 1997, 1984) and consists of the following nine subscales: Identity (17 items), Timeline-Acute/Chronic (6 items), Timeline-Cyclical (4 items), Consequences (6 items), Personal Control (6 items), Treatment Control (5 items), Illness Coherence (5 items), Emotional Representations (6 items), and Causes (21 items). Higher scores on the Timeline-Acute/Chronic, Timeline-Cyclical, Consequences, and Emotional Representations subscales indicate higher perceived chronicity of ASD, increased unpredictability of ASD symptoms, greater harmful consequences from ASD, and more negative feelings about ASD, respectively. Additionally, higher scores on the Personal Control, Treatment Control, and Illness Coherence subscales indicate greater perceived caregiver control of ASD symptoms, hopefulness regarding treatments for ASD, and confidence in personal understanding of ASD. The Identity and Cause subscales cover observed symptoms and possible causes of ASD. Neither the Identity nor Cause subscales were used in this study, as both the scoring and purposes of these subscales differ from that of the other subscales, and the validation of the factor structure of the IPQ-R-ASD was done without their inclusion. Subscale internal consistencies of the IPQ-R-ASD range from α = 0.80 (Timeline-Cyclical) to α = 0.87 (Timeline-Acute/Chronic; Mire et al., 2017). The Treatment Control and Personal Control subscales were collapsed into a single Control subscale for the purposes of this study considering items from both subscales loaded onto a single factor in prior studies examining the factor structure of the IPQ-R-ASD (Mire et al., 2017). Further, a CFA with this study’s sample supported this 6-factor solution with a single Control factor, χ^2^(512) = 1451.04, *p* ≤ 0.001; CFI = 0.94; TLI = 0.93; RMSEA = 0.08; and SMRM = 0.09, and the internal consistency of the collapsed Control subscale (α = 0.87) was higher than the internal consistency estimates of each subscale separately (Personal Control subscale: α = 0.82; Treatment Control subscale: α = 0.76). In our sample, overall internal consistencies for the six IPQ-R-ASD subscales (i.e., Timeline-Acute/Chronic, Timeline-Cyclical, Consequences, Control, Illness Coherence, Emotional Representations) were consistent with past studies (ranging from α = 0.82 to α = 0.92) and subscale scores were calculated by summing the items comprising each subscale.

The Coping Orientation of Problems Experienced Inventory (Brief-COPE; Carver, 1997) is a 28-item self-report questionnaire that was used to assess coping behaviors in response to stressful life events. The Brief-COPE is a shortened version of the original Coping Orientation of Problems Experienced Inventory (Carver et al., 1989). Each question is answered on a 4-point Likert scale ranging from 1 (“I haven’t been doing this at all”) to 4 (“I’ve been doing this a lot”). In samples of children with ASD, the Brief-COPE has been found to have a four-factor structure (Hastings et al., 2005) derived from 14 scales comprising the original measure: a 9-item Active-Avoidance subscale (i.e., all items from substance use, behavioral disengagement, self-blame, venting and emotions scales, 1 item from distraction scale), a 7-item Problem-Focused subscale (i.e., all items from planning, active coping, instrumental social support scales, 1 item from emotional support scale), a 6-item Positive Coping subscale (i.e., all items from humor and positive reframing scales, 1 item from acceptance and emotional support scales), and a 6-item Religious/Denial Coping subscale (i.e., all items from the religious and denial scales). For the purposes of this study, only the Problem-Focused Coping subscale (α = 0.81) and the Active-Avoidance Coping subscale (α = 0.87) of the Brief-COPE were used as the other two subscales had poor internal consistencies (α’s = 0.51 and 0.59) in our sample. Subscale scores for the Brief-COPE were calculated by averaging the items comprising each subscale.

The Treatment Behaviors for Child’s ASD (adapted from Dardennes et al., 2011; Mire et al., 2017) was used to obtain information about caregiver intentions to seek or continue to seek supportive services for their children’s symptoms of ASD. Caregivers were asked to indicate whether their children are currently receiving any supportive services for their symptoms of ASD by selecting “yes” or “no.” If caregivers responded in the affirmative, they were then asked to indicate the type(s) of supportive services their children are currently receiving by making selections from predetermined treatment categories (e.g., behavior therapy, psychotropic medication, speech/language therapy, occupational therapy). Caregivers were also asked how likely they are to seek additional supportive services for their children’s symptoms of ASD in the next month on a 7-point Likert scale ranging from 1 (Very Unlikely) to 7 (Very Likely) and were then asked to indicate (from predetermined treatment categories) the type(s) of supportive services they intend to seek. If caregivers indicated that their children are not currently receiving supportive services for their ASD, then caregivers were asked to indicate how likely they are to seek supportive services for their children’s symptoms of ASD in the next month on a 7-point Likert scale ranging from 1 (Very Unlikely) to 7 (Very Likely) and were then asked to indicate (from predetermined treatment categories) the type(s) of services they intend to seek. For this study, treatment seeking behaviors was operationalized as caregivers’ intentions to seek treatment or to continue to seek treatment for their children’s symptoms of ASD in the next month. A treatment seeking behavior variable was calculated by merging caregivers’ responses from item 3 (i.e., intentions to continue with treatment services in the next month) and item 7 (i.e., intentions to seek treatment services in the next month) into a single “intentions to seek treatment” variable. This approach was taken because participants only answered one of the two questions based on whether their children had received services in the past.

### 2.3. Procedures

Institutional Review Board (IRB) approval was obtained from the university through which this study was conducted prior to data collection. Informed consent was received from all participants prior to their involvement in study procedures. At in-person recruitment sites, caregivers were provided with a flyer that contained a link to the study survey if a provider at the recruitment site had confirmed a diagnosis of ASD for the caregiver’s child. Participants were incentivized to complete the study through entry into a raffle to win one of eight $50 Amazon gift cards or receive a $5 Amazon gift card. For participants recruited through Prolific, a 5-item screener developed for this study (i.e., Childhood Health Services Survey) was used to identify caregivers of autistic children by asking them about their children’s health status and use of services. Participants received $0.20 for their completion of the screener. If participants had a child diagnosed with ASD based on their responses to the screener, they were invited to participate in the study and were paid an additional $5.00 for their participation. Further, caregivers recruited from Prolific were asked to endorse on the Identity subscale of the IPQ-R-ASD measure how many behaviors consistent with ASD were currently present for their children with 95% of participants (N = 265) observing 6 or more ASD symptoms; a cut-off established in prior work as being consistent with an ASD diagnosis (Al Anbar et al., 2010). Study measures were administered in the following order via an online survey platform (i.e., Qualtrics): a brief demographic form, the IPQ-R-ASD, the Brief-COPE, and the Treatment Behaviors for Child’s ASD.

### 2.4. Data Analytic Plan and Preliminary Analyses

Data were analyzed using IBM SPSS Version 29 (IBM Corp, 2023) and MPLUS Version 8.10 (L. K. Muthén & Muthén, 2017). Prior to testing the study hypotheses, a series of preliminary analyses were run to ensure no assumptions of the planned statistical tests were violated and that no variables had out-of-range values. Univariate outliers were handled by determining whether they were plausible values and if they were identified as data entry errors, they were replaced with the next highest or lowest non-outlying value; no univariate outliers were identified for our study variables. The Mahalanobis distance formula identified three participants whose data contained a multivariate outlier, so models were run with and without their inclusion (Leys et al., 2018). The percentage of missing data for demographic and study variables ranged from 0 to 1.7%. Given that a missing data percentage of 10% or less is considered inconsequential, no method to handle missing data for demographic or study variables was used besides pairwise deletion or WLS-based estimators for ordinal variables (Bennett, 2001; Enders & Bandalos, 2001; B. Muthén et al., 1987).

Correlations were run to examine associations between the study variables and to identify caregiver- or child-specific demographic variables that should be included as potential covariates in planned analyses. See Table 3 for descriptive statistics and correlations among study variables. As length of time since diagnosis and ASD severity level were significantly associated with treatment seeking behaviors, they were included as covariates in relevant models. To test the hypothesis that cognitive and emotional illness perceptions are associated with caregivers’ treatment seeking behaviors through both Problem-Focused Coping and Active-Avoidance Coping, a path analysis was run consisting of six exogenous (predictor) variables (IPQ-R-ASD subscales: Consequence, Control, Emotional Representation, Illness Coherence, Timeline-Cyclical, and Timeline-Acute/Chronic), two mediating variables (Problem-Focused Coping and Active-Avoidance Coping), one endogenous (outcome) variable (treatment seeking behaviors), and two covariates (length of time since diagnosis and ASD severity). The significance of indirect effects was tested using the delta method (i.e., bootstrapping with 1000 samples) and by calculating bias-corrected confidence intervals.

## 3. Results

**Figure 1:**
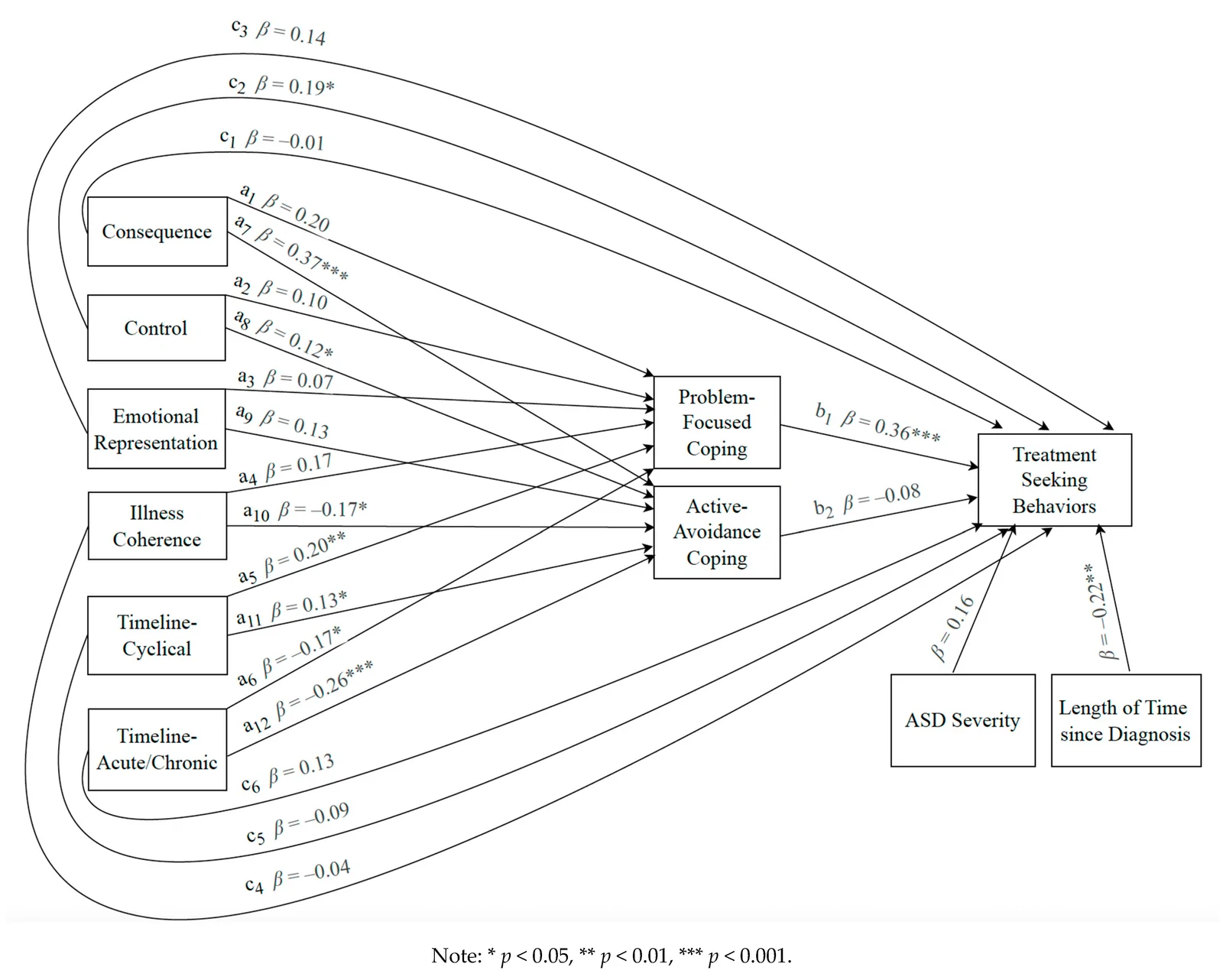
Path analysis of CSM with direct effects.

**Figure 2:**
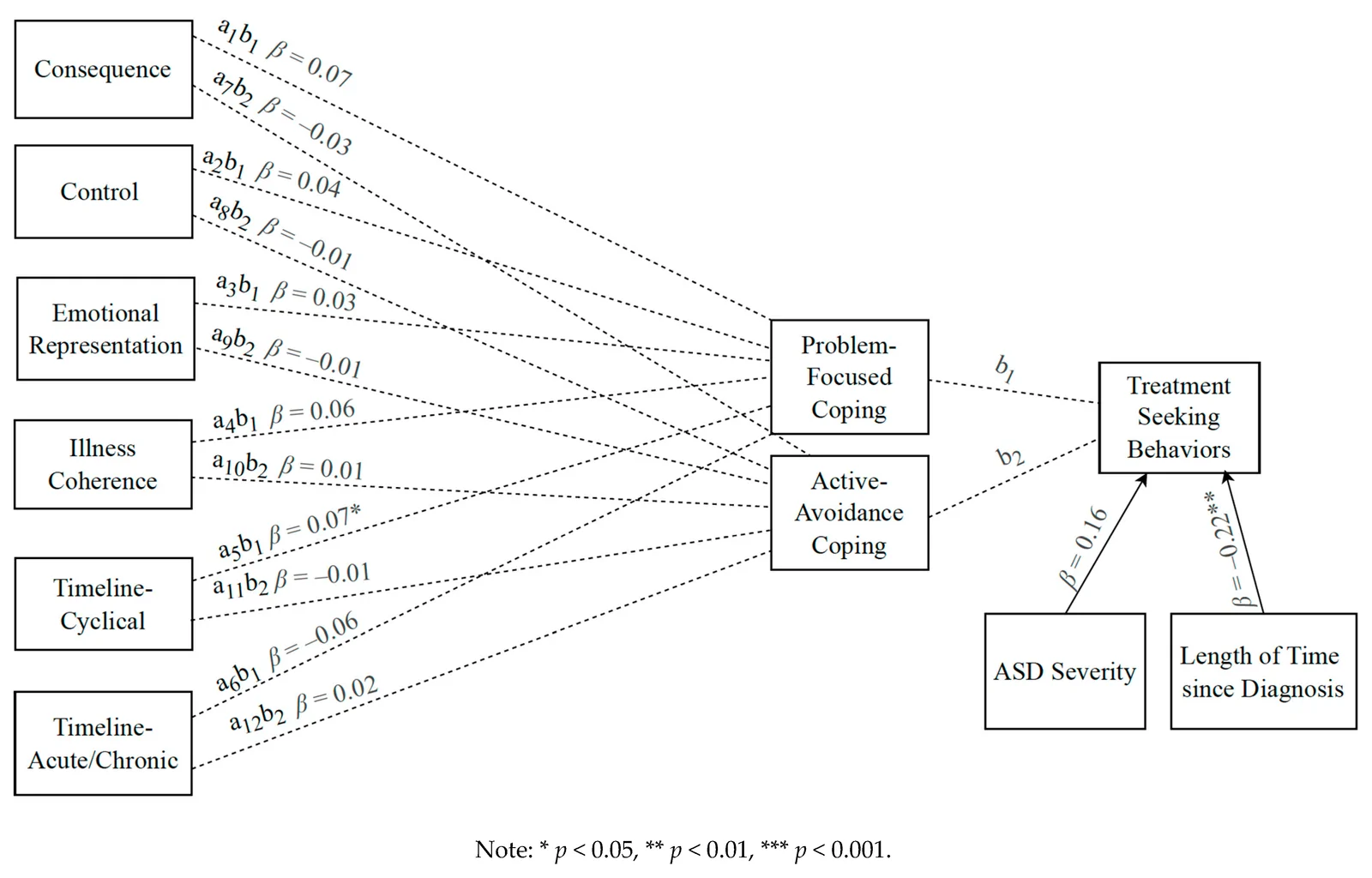
Path analysis of CSM with indirect effects.

The hypothesized model was an excellent fit to the data as evidenced by the following fit indices: χ^2^(5) = 4.36, *p* = 0.50, RMSEA < 0.001, CFI = 1.00, TLI = 1.04, WRMR = 0.34. See Figure 1 for a graphical depiction of this model. Results revealed significant direct effects of Timeline-Cyclical and Timeline-Acute/Chronic on Problem-Focused Coping (Path a_5_: β = 0.20, *p* = 0.005, 95% CI [0.07, 0.39]; Path a_6_: β = −0.17, *p* = 0.03, 95% CI [−0.27, −0.02]) and a significant direct effect of Problem-Focused Coping on treatment seeking behaviors (Path b_1_: β = 0.36, *p* < 0.001, 95% CI [0.23, 0.48]). All except one illness perception had significant direct effects on Active-Avoidance Coping (Consequence Path a_7_: β = 0.37, *p* < 0.001, 95% CI [0.22, 0.51]; Control Path a_8_: β = 0.12, *p* = 0.03, 95% CI [0.03, 0.34]; Illness Coherence Path a_10_: β = −0.17, *p* = 0.04, 95% CI [−0.38, −0.03]; Timeline-Cyclical Path a_11_: β = 0.13, *p* = 0.01, 95% CI [0.04, 0.34]; Timeline-Acute/Chronic Path a_12_: β = −0.26, *p* < 0.001, 95% CI [−0.43, −0.13]); however, there was not a significant direct effect of Active-Avoidance Coping on treatment seeking behaviors (Path b_2_: β = −0.08, *p* = 0.39, 95% CI [−0.22, 0.05]). Of the illness perceptions, only Control had a significant direct effect on treatment seeking behaviors (Path c_2_: β = 0.19, *p* = 0.03, 95% CI [0.06, 0.34]). The indirect effect from Timeline-Cyclical to treatment seeking behaviors through Problem-Focused Coping was significant (Path a_5_b_1_: β = 0.07, *p* = 0.02, 95% CI [0.01, 0.13]). However, the indirect effects for the other illness perceptions (i.e., Consequence, Control, Emotional Representation, Timeline-Acute/Chronic, Illness Coherence) to treatment seeking behaviors through Problem-Focused Coping or Active-Avoidance Coping were not. Figure 2 illustrates all indirect paths from illness perceptions to treatment seeking behaviors through both Problem-Focused Coping and Active-Avoidance Coping and Table 4 reports all parameter estimates and confidence intervals for these indirect effects. The total effect (i.e., the sum of both direct and indirect effects) of Control on treatment seeking behavior through Problem-Focused Coping was significant (β = 0.21, *p* < 0.01, 95% CI [0.12, 0.31]). No other total effects within the model were significant. Finally, the direct effect of length of time since diagnosis to treatment seeking behavior was significant (β = −0.21, *p* = 0.003, 95% CI [−0.42, −0.03]) whereas the direct effect of ASD severity to this outcome was not (β = 0.16, *p* = 0.06, 95% CI [−0.02, 0.34]).

## 4. Discussion

The primary objective of this study was to build upon recent studies that have examined select parts of the CSM (Al Anbar et al., 2010; Dardennes et al., 2011) in caregivers of autistic children by examining all major components included in the theory (i.e., illness perceptions, coping behaviors, and illness outcomes). It is our intent that the results of this study will be used to inform best practices when working with caregivers of autistic children so that factors impacting the decision-making of caregivers, particularly caregivers’ intentions about whether they will seek supportive services for their children, are better understood.

Overall, our findings partially supported the CSM, as Problem-Focused Coping mediated the association between caregivers’ tendency to view their children’s ASD symptoms as unpredictable or “coming and going” (i.e., Timeline-Cyclical) and their intentions to seek (or to continue to seek) treatment for their children. Although past studies from the chronic illness literature have shown that associations between the unpredictable nature of chronic illnesses and other negative illness outcomes (i.e., psychological distress, anxiety) are explained by maladaptive coping strategies (e.g., Hagger et al., 2017), this illness perception has also been shown to be positively linked to evidence-based treatments (i.e., psychopharmaceutical treatments) in a sample of caregivers of autistic children (Al Anbar et al., 2010). Given this link and our finding that caregivers’ tendency to view their children’s symptoms as unpredictable (Timeline-Cyclical) is significantly and positively related to both forms of coping (i.e., Problem-Focused Coping and Active-Avoidance Coping), it is not surprising that the only significant pathway to treatment seeking behaviors was through Problem-Focused Coping. It may be that, as Leventhal et al. (1980) proposed, when caregivers perceive their children’s ASD symptoms to vary greatly day-to-day and then actively think of steps or strategies to help with this unpredictability, their intentions to seek treatment for their children increase. In contrast, avoidance-based coping (e.g., giving up trying to cope, self-blaming or self-criticizing, engaging in distractions) would be less conducive to taking action to improve their children’s daily functioning when compared to a problem-solving-based approach. Indeed, the process of thinking through specific behavioral plans (e.g., “If X happens, then I will do Y”) has been shown to be related to actual behavioral outcomes (Baumeister et al., 2011), so the planning that accompanies Problem-Focused Coping may be a better facilitator of action-oriented outcomes relative to avoidance-based coping strategies. Alternatively, the uncertainty associated with viewing symptoms as unpredictable may allow caregivers to be more open to the idea that a positive change in their children’s symptoms is possible. When parents perceive ASD symptoms as dynamic (e.g., having easier versus more challenging days) rather than fixed, this may foster a sense of hope that overall improvement is a tangible goal and may prompt them to pursue solutions or social support (i.e., types of Problem-Focused Coping). In turn, this optimism and hope regarding potential symptom change combined with more positive coping strategies may increase intentions or motivation to seek treatment.

Contrary to our predictions, caregiver coping behaviors did not mediate the path between any other cognitive or emotional illness perceptions and treatment seeking behaviors in this sample. It is possible that the tendency to view ASD symptoms as unpredictable is the most salient and challenging illness perception for caregivers of autistic children and requires some form of coping behavior; a notion supported by its stronger and positive associations with both forms of coping relative to other illness perceptions. This perception coupled with active coping strategies, which may be adopted because of encouragement or support from others or through therapeutic services, appears to translate into a greater likelihood of treatment seeking behaviors. We also found that the perception that autism is a chronic condition (i.e., Timeline-Acute/Chronic) is negatively associated with both forms of coping, the perception that autism is understandable (i.e., Illness Coherence) is negatively associated with Active-Avoidance Coping, and the perceptions that autism is controllable (i.e., Control) and yields harmful consequences (i.e., Consequences) are positively associated with Active-Avoidance Coping. Such results suggest that psychoeducation about ASD and providing alternative and more adaptive strategies for caregivers may be beneficial to their psychological well-being, so they are better equipped to meet the needs of their children.

Another potential reason why the CSM model is only partially supported in this sample may be because of how treatment seeking behaviors were conceptualized and measured in this study. Our outcome variable assessed caregivers’ intentions to seek (or to continue to seek) treatment over the next month; however, this variable does not account for the influence that past treatment seeking behaviors may have on current intentions. In this study, the length of time since diagnosis was significantly and positively associated with caregiver intentions to seek treatment, yet this variable does not capture caregivers who do not seek treatment because their children no longer need services or who recognize recommended treatments are unavailable. For these caregivers, a lack of treatment seeking could simply be due to no longer needing services or not having access to services rather than having maladaptive perceptions about ASD or using maladaptive coping behaviors. In this instance, other autism-related outcomes, such as maintenance of treatment gains (e.g., child social functioning, child use of adaptive skills) may be a more relevant outcome to consider when examining relations with ASD-specific perceptions and coping behaviors.

Although prior studies have linked certain illness perceptions with specific treatment-related outcomes (e.g., applied behavioral analysis, psychopharmaceutical treatments; Al Anbar et al., 2010), our findings revealed that, out of all illness perceptions, only caregivers’ perceived control over their children’s ASD symptoms had a direct effect on treatment seeking behaviors even when taking into account the length of time since diagnosis and ASD symptom severity. In other words, caregivers who perceive greater control over their children’s ASD symptoms are more likely to seek (or continue to seek) treatment and this effect is not explained by caregivers’ coping behaviors. This finding is not aligned with the coping mediation effect proposed in the CSM, but it does suggest that personal and treatment control over ASD symptoms may be a driving force behind caregivers’ intentions to seek supportive services for their autistic children. These findings are consistent with a meta-analysis by Hagger et al. (2017) in that perceived control over a health concern was found to be predictive of adaptive health outcomes. Given cognitions are considered malleable (Moreau, 2022), our findings have important implications for providers who work with caregivers of autistic children, as they are in a unique position to help caregivers alter their inaccurate or unhelpful cognitions using a CBT-based framework that could then promote treatment seeking behaviors (Fréchette-Simard et al., 2018).

### 4.1. Clinical Implications

The results of this study have provided useful information about potential factors that may influence treatment seeking behaviors in caregivers of autistic children and is highly relevant for providers who work with caregivers of autistic children, especially when assisting them in navigating the complexities of obtaining services for their children. Arguably, providers are the most influential to caregivers when their children initially receive an ASD diagnosis, as this may be the first time caregivers interact with and discuss their children’s symptoms with a provider. It also marks a pivotal point when children become eligible for services that would otherwise not be available to them without an ASD diagnosis (Malik-Soni et al., 2022). While there are a few aspects of an autism feedback session that are generally recommended according to published guidelines (i.e., provide information regarding the diagnostic process; autism etiology, symptoms and presentation; referrals for support; Pattison et al., 2022), the findings from this study suggest that assessing caregiver-specific variables (i.e., thoughts about children’s ASD diagnosis, strategies used to cope with difficult ASD-related behaviors) at this stage in a family’s autism journey may be beneficial. In fact, providers who allot time to discuss these factors during a diagnostic feedback session may be better equipped to recommend educative supports that best fit the family’s needs. Additionally, providers who have ongoing contact with caregivers should continue to assess these caregiver-specific factors over time considering caregivers’ knowledge about ASD and their children’s unique behaviors will evolve as they engage with new providers and resources. It is also possible that some of the information caregivers are exposed to is inaccurate, which could influence their ASD perceptions in a counterproductive manner. However, time constraints for providers during feedback sessions should be acknowledged so providers are encouraged to implement strategies that are the most relevant and will presumably have the greatest impact for families.

Systematic and meta-analytic reviews have shown the benefits of CBT-based strategies in adults and children with chronic illness (Tao et al., 2023; Thompson et al., 2011). In fact, there is emerging support that cognitive restructuring and psychoeducation elements of CBT improve medical adherence, physical and emotional distress, physiologic indicators of illness, and communication skills. There is also some evidence to suggest that caregiver-focused interventions using CBT-based approaches have positive effects on certain psychosocial outcomes in caregivers of autistic children (Yu et al., 2019). As such, providers should consider employing CBT-based strategies (e.g., Traeger, 2020) to alter any maladaptive or unhelpful thoughts shared by caregivers starting as early as the diagnostic assessment feedback session and continuing throughout their involvement with caregivers. For instance, providers could offer brief support using cognitive restructuring based on psychoeducation to increase caregivers’ perceptions of controllability with the goal of either mobilizing initial treatment seeking or continuing treatment seeking if necessary. Providers may also recommend or model more adaptive, problem-focused coping strategies if maladaptive coping behaviors are disclosed in an effort to influence treatment seeking behaviors. For example, in addition to discussing treatment options for a family whose child was recently diagnosed with ASD, providers could assist caregivers in identifying providers in their area and specific school personnel with whom they should connect. This modeling of a step-by-step approach for caregivers may help minimize how unsupported many caregivers feel after their children receive an ASD diagnosis (Crane et al., 2016) and give them a better understanding of how to proceed post-diagnosis, which is sometimes unclear for caregivers (Keenan et al., 2010). As these suggested applications of CBT-based strategies were not explicitly evaluated by this study, further work is needed to empirically validate their use for these purposes. Further, these recommendations are tied to the sampled population and may not be generalizable to other caregiver populations. Ultimately, the assessment and modification of certain caregiver-specific factors as early as possible may be beneficial in facilitating early access to treatment, which may lead to better developmental gains and long-term outcomes for autistic children (Clark et al., 2017; Dawson et al., 2012; Vivanti & Dissanayake, 2016).

### 4.2. Limitations and Future Directions

Although this is the first known study to examine all components of the CSM in a sample of caregivers of autistic children, there are also some limitations that warrant discussion. First, the primary recruitment method used for this study (i.e., crowd-sourcing website) resulted in a sample of caregivers who are predominantly White, women, married, college-educated, English-speaking, and from middle-income households, which is only representative of a subset of caregivers with autistic children in the US. As a result, the conclusions drawn concerning caregivers’ perceptions, coping behaviors and treatment seeking intentions related to their children’s ASD diagnosis are likely not applicable to caregivers of autistic children from different backgrounds or lived experiences and more diverse samples are needed to evaluate the CSM. Relatedly, the act of simply volunteering for this study may suggest caregivers hold certain perceptions of ASD (e.g., more controllable, motivated to seek answers), which should also be considered when interpreting these results. Second, there was no way to verify the reported ASD diagnosis of children for those participants who were recruited via Prolific. Third, these data were collected from caregivers at one timepoint, which was then used to draw conclusions about a theory involving processes that theoretically unfold over time. As such, the cross-sectional nature of this study precludes inferences of causality among the constructs of interest. Another drawback of collecting data at one timepoint is that our outcome measure only captured intentions to seek treatment in the next month, so there was no way of assessing whether these services were ever received. Future studies should consider employing longitudinal data collection methods (e.g., cross-lagged study design or time series analysis) to better understand how these specific constructs may change and influence each other over time and whether these changes differ based on caregiver and child characteristics. Fourth, all data captured in this study were based on caregiver self-report, which may artificially inflate associations found between study variables simply due to shared-method variance. It should be noted that different results may have been obtained if treatment seeking behaviors were operationalized as actual services sought or received rather than treatment seeking intentions, which is a possibility worthy of future investigation. Fifth, caregivers, on average, reported that their children were diagnosed with ASD 4.5 years ago (range = 0 to 15 years) and given the long-term positive impact early intervention has for some autistic children, it would be important for future studies to examine perceptions, coping, and treatment seeking behaviors closer to when children first receive an ASD diagnosis (i.e., at the time of or shortly after diagnostic feedback), as this is a critical period when providers work with caregivers to navigate intervention options for their children. It is also worth mentioning that only 40% of participants who were recruited through private practices (compared to 80% of participants recruited through Prolific) completed the entirety of this study’s survey, which may be related to the proximity to when caregivers received an ASD diagnosis for their children and an artifact of how caregivers were coping in the moment. To allow caregivers time to process the diagnosis while increasing measure completion rates, future studies should consider determining caregiver interest in study participation at the feedback appointment and then follow-up with interested caregivers one or two weeks after this appointment. By comparison, participants recruited via Prolific are experienced at online surveys and expect compensation for their time, which may inflate completion rates and introduce a sampling bias so they might not be representative of typical caregivers navigating the complexities of an ASD diagnosis for their children. Finally, it will be important for other potential mediators to be examined to better understand the influence of ASD-related perceptions on treatment seeking behaviors, such as the availability of services, caregiver trust of providers, and caregiver values (i.e., culture-specific values and preferences), as these variables have been shown to be linked with specific types of treatments that caregivers select for their children (Saez et al., 2023).

## 5. Conclusions

Although the results of this study only partially supported the CSM in a sample of caregivers of autistic children, we did find that problem-focused coping mediated the positive association between caregiver perceptions about the unpredictability of ASD symptoms and treatment seeking behaviors and caregiver perceptions about the controllability of ASD symptoms had a direct effect on treatment seeking behaviors. These findings have important considerations for providers when working with caregivers of autistic children, such as assessing and potentially modifying aspects of caregivers’ perceptions and coping behaviors during ASD feedback sessions as a way to promote prompt treatment seeking for autistic children. Future studies should consider replicating these findings using longitudinal study designs and measuring these constructs of interest within close proximity of the initial diagnosis. Finally, other variables (e.g., access to services, trust in provider, and caregiver cultural values) shown to be related to treatment outcomes should be explored as potential mediators within the CSM model when applied to caregivers of autistic children.

## Figures and Tables

**Table 1:** Caregiver demographic characteristics.

Characteristic	M (SD)	Range (Years)
Caregiver Age	40.67 (9.19)	22–69
	**N (%)**	
Caregiver Self-Reported Gender Identity		
Woman	180 (63.4)	
Man	95 (33.5)	
Non-binary	9 (3.2)	
Caregiver Race/Ethnicity		
American Indian/Indigenous American/Alaskan Native	1 (0.4)	
Native Hawaiian/Other Pacific Islander	1 (0.4)	
Asian American	14 (4.9)	
Hispanic/Latino(a)/Spanish Origin	19 (6.7)	
African American/Black American	43 (14.8)	
White	201 (70.8)	
College-Educated	184 (64.1)	
Caregiver Marital Status		
Married/Partnered	210 (73.9)	
Single	38 (13.4)	
Divorced/Separated	21 (7.4)	
Widowed	7 (2.5)	
Caregiver Employment		
Full-time	187 (65.8)	
Part-time	45 (15.8)	
Unemployed or Disabled	52 (18.3)	
Caregiver Relation to Child		
Biological Parent	254 (89.4)	
Caregiver Income		
$20,000 or less	18 (6.3)	
$21,000 to $50,000	74 (26)	
$51,000 to $100,000	113 (39.8)	
$101,000 or more	76 (26.8)	

**Table 2:** Child demographic characteristics and their diagnostic and treatment information.

Characteristic	M (SD)	Range (Years)
Child Age	9.74 (4.47)	2–17
Child Age at First Concern	35 months (29)	0–180 months
Child Age at Diagnosis	5.16 (2.87)	1–15
Total Hours of Weekly Treatment	11.12 (11.48)	0–50
	**N (%)**	
Child Gender Identity (Caregiver Reported)		
Boy	195 (68.7)	
Girl	76 (26.8)	
Transgender	6 (2.1)	
Non-binary	4 (1.4)	
Child Race/Ethnicity		
American Indian/Indigenous American/Alaskan Native	3 (1.0)	
Asian American	11 (3.9)	
Hispanic/Latino(a)/Spanish Origin	13 (4.6)	
African American/Black American	36 (12.7)	
More than 1 Ethnicity Reported	36 (12.7)	
White	176 (62)	
Expert Diagnostician		
Psychologist	139 (48.9)	
Pediatrician/Physician/Nurse Practitioner	124 (43.7)	
Length of Time Since Diagnosis		
0–2 years	117 (41.8%)	
3–6 years	79 (28.2%)	
7–15 years	84 (30.0%)	
Child ASD Severity Specification		
Level 1 (Requiring Support)	116 (40.8)	
Level 2 (Requiring Substantial Support)	91 (32.0)	
Level 3 (Requiring Very Substantial Support)	31 (10.9)	
Comorbid Intellectual Developmental Disability	81 (28.5)	
Child Language Ability		
Preverbal or Nonspeaking	25 (8.8)	
Babbling	18 (6.3)	
Single Words Only	17 (6.0)	
Phrase Speech	50 (17.6)	
Fully Verbal	173 (60.9)	
Current Receipt of Treatment	215 (75.7%)	
Continuation of Treatment	197 (69.5%)	
Treatments Received		
Behavior therapy (e.g., Applied Behavior Analysis)	108 (38.0%)	
Social skills training	95 (33.5%)	
School-based speech/language therapy	95 (33.5%)	
School-based occupational therapy	78 (27.5%)	
Private speech/language therapy	64 (22.5%)	
Cognitive-behavioral therapy	62 (21.8%)	
Psychotropic mediation	62 (21.8%)	

**Table 3:** Descriptives and correlations among study variables and covariates.

Variables	1	2	3	4	5	6	7	8	9	10	11
1. Timeline-A/C (IPQ)	--										
2. Consequences (IPQ)	0.29***	--									
3. Control (IPQ)	−0.16*	−0.03	--								
4. Emotional Rep (IPQ)	−0.09	0.57***	−0.01	--							
5. Illness Coh (IPQ)	0.18**	−0.33***	0.13*	−0.55***	--						
6. Timeline-C (IPQ)	−0.14**	0.32***	0.15*	0.44***	−0.35***	--					
7. Avoidance (COPE)	−0.26***	0.41***	0.09	0.46***	−0.43***	0.38***	--				
8. Problem-Focused (COPE)	−0.11	0.22***	0.20***	0.21***	−0.09	0.27***	0.24***	--			
9. Treatment Seeking Bxs	0.04	0.18**	0.21***	0.15*	−0.09	0.09	0.07	0.37***	--		
10. Time Since Diagnosis	0.15*	0.16**	0.02	0.09	0.03	−0.03	0.04	0.08	−0.14*	--	
11. ASD Severity	0.19**	0.39***	0.04	0.22***	−0.17*	0.10	0.08	0.08	0.20**	−0.02	--
Mean (SD)	20.84 (4.93)	19.33 (5.31)	25.40 (3.55)	16.87 (5.68)	17.74 (4.45)	11.11 (3.71)	13.94 (5.08)	15.46 (4.12)	5.56 (1.91)	4.57 (3.94)	1.64 (0.71)

Note: Timeline-A/C = Timeline-Acute/Chronic; Emotional Rep = Emotional Representation; Illness Coh = Illness Coherence; Timeline-C = Timeline-Cyclical; Avoidance = Active-Avoidance Coping; Problem-Focused = Problem-Focused Coping; Bxs = Behaviors; IPQ = Illness Perception Questionnaire–Revised–ASD; COPE = Brief Coping Orientation of Problems Experienced Inventory. * *p* < 0.05, ** *p* < 0.01, *** *p* < 0.001.

**Table 4:** Parameter estimates and confidence intervals of indirect effects.

Indirect Effect	Path Notation	β	*p*	95% CI
Consequence → __ → Treatment Seeking Bxs				
Problem-Focused Coping	a_1_b_1_	0.07	0.09	−0.01, 0.15
Active-Avoidance Coping	a_7_b_2_	−0.03	0.40	−0.10, 0.04
Control → __ → Treatment				
Problem-Focused Coping	a_2_b_1_	0.04	0.24	−0.02, 0.09
Active-Avoidance Coping	a_8_b_2_	−0.01	0.57	−0.04, 0.02
Emotional Rep → __ → Treatment Seeking Bxs				
Problem-Focused Coping	a_3_b_1_	0.03	0.48	−0.04, 0.09
Active-Avoidance Coping	a_9_b_2_	−0.01	0.57	−0.04, 0.02
Illness Coherence → __ → Treatment Seeking Bxs				
Problem-Focused Coping	a_4_b_1_	0.06	0.11	−0.01, 0.13
Active-Avoidance Coping	a_10_b_2_	0.01	0.43	−0.02, 0.05
Timeline-Cyclical → __ → Treatment Seeking Bxs				
Problem-Focused Coping	a_5_b_1_	0.07	0.02	0.01, 0.13
Active-Avoidance Coping	a_11_b_2_	−0.01	0.43	−0.04, 0.02
Timeline-Acute/Chronic → __ → Treatment Seeking Bxs				
Problem-Focused Coping	a_5_b_1_	−0.06	0.06	−0.13, 0.003
Active-Avoidance Coping	a_12_b_2_	0.02	0.41	−0.03, 0.07

Note: 95% CI = 95% confidence interval, lower and upper limits separated by commas; Rep = Representation; Bxs = Behaviors.

## Data Availability

The raw data supporting the conclusions of this article will be made available by the authors on request.

## Abbreviations

The following abbreviations are used in this manuscript:

CSM	Common Sense Model
ASD	Autism Spectrum Disorder
CBT	Cognitive-Behavioral Therapy
APA	American Psychiatric Association
IPQ-R	Illness Perception Questionnaire–Revised
Brief-COPE	Brief Coping Orientation of Problems Experienced Inventory
CI	Confidence Interval
RMSEA	Root Mean Square Error of Approximation
CFI	Comparative Fit Index
TLI	Tucker–Lewis Index
WRMR	Weighted Root Mean Square Residual
